# Correction: Hematological and biochemical alterations in preeclampsia: Readings from cord blood analysis

**DOI:** 10.1371/journal.pone.0344715

**Published:** 2026-03-10

**Authors:** Ahmed Abu Siniyeh, Mohammad Alsahori, Talal Al Qaisi, Majed Al-Holi

The second author’s name is spelled incorrectly. The correct name is: Mohammad Alsahori. The correct citation is: Siniyeh AA, Alsahori M, Al Qaisi T, Al-Holi M (2025) Hematological and biochemical alterations in preeclampsia: Readings from cord blood analysis. PLoS One 20(5): e0324460. https://doi.org/10.1371/journal.pone.0324460.
