# Supplementary figures and images for: Hematological and biochemical alterations in preeclampsia: Readings from cord blood analysis
Source: PLoS One. 2025 May 30;20(5):e0324460. doi: 10.1371/journal.pone.0324460 (PMC12124850; doi:10.1371/journal.pone.0324460)

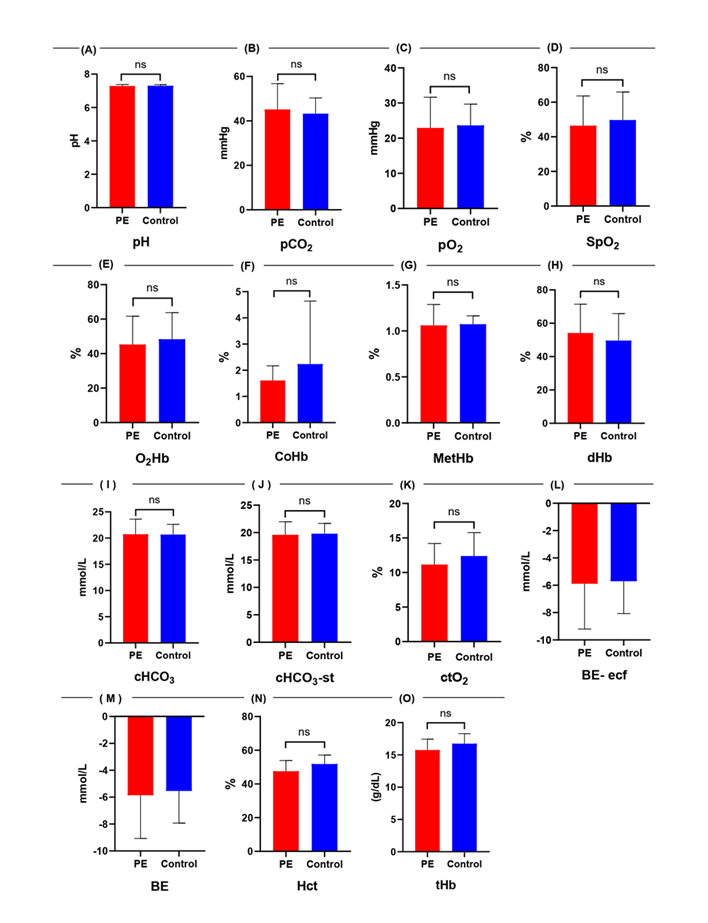

Supplement: S1 Fig — The graphs present various parameters of cord blood gas in control group vs preeclampsia group. The graphs illustrate the following (A) pH levels (B) Partial pressure of carbon dioxide (pCO₂). (C) Partial pressure of oxygen (pO₂). (D) Oxygen saturation (SpO₂). (E) Oxyhemoglobin (O₂Hb). (F) Carboxyhemoglobin (COHb). (G) Methemoglobin (MetHb). (H) Deoxygenated hemoglobin (dHb). (I) Concentration of bicarbonate (cHCO₃). (J) Standardized bicarbonate (cHCO₃-st). (K) Total oxygen content (ctO₂). (L) Base excess in extracellular fluid (BE-ecf). (M) Base excess (BE). (N) Hematocrit (Hct). (O) Total hemoglobin (tHb). Significant findings are indicated by asterisks (*) for statistically significant differences, while “ns” denotes non-significant results. (TIF) [file pone.0324460.s003.tif]

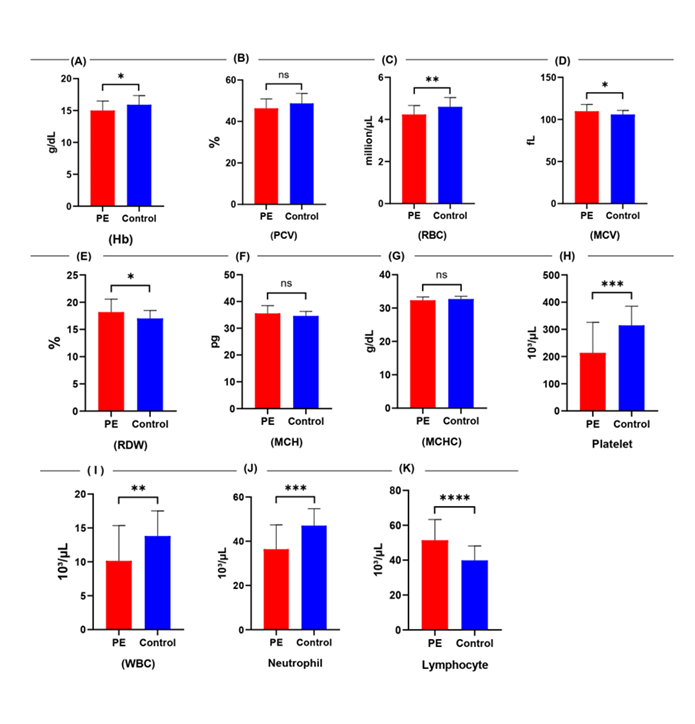

Supplement: S2 Fig — This figure displays various complete blood count (CBC) parameters measured in a control group and preeclampsia group. Panels (A) through (K) illustrate the following hematological parameters: (A) hemoglobin (Hb) levels, (B) packed cell volume (PCV), (C) red blood cell (RBC) count, (D) mean corpuscular volume (MCV), (E) red cell distribution width (RDW), (F) mean corpuscular hemoglobin (MCH), (G) mean corpuscular hemoglobin concentration (MCHC), (H) platelet count, (I) white blood cell (WBC) count, (J) neutrophil count, and (K) lymphocyte count. Significant findings are indicated by asterisks (*) for statistically significant differences, while “ns” denotes non-significant results. (TIF) [file pone.0324460.s004.tif]

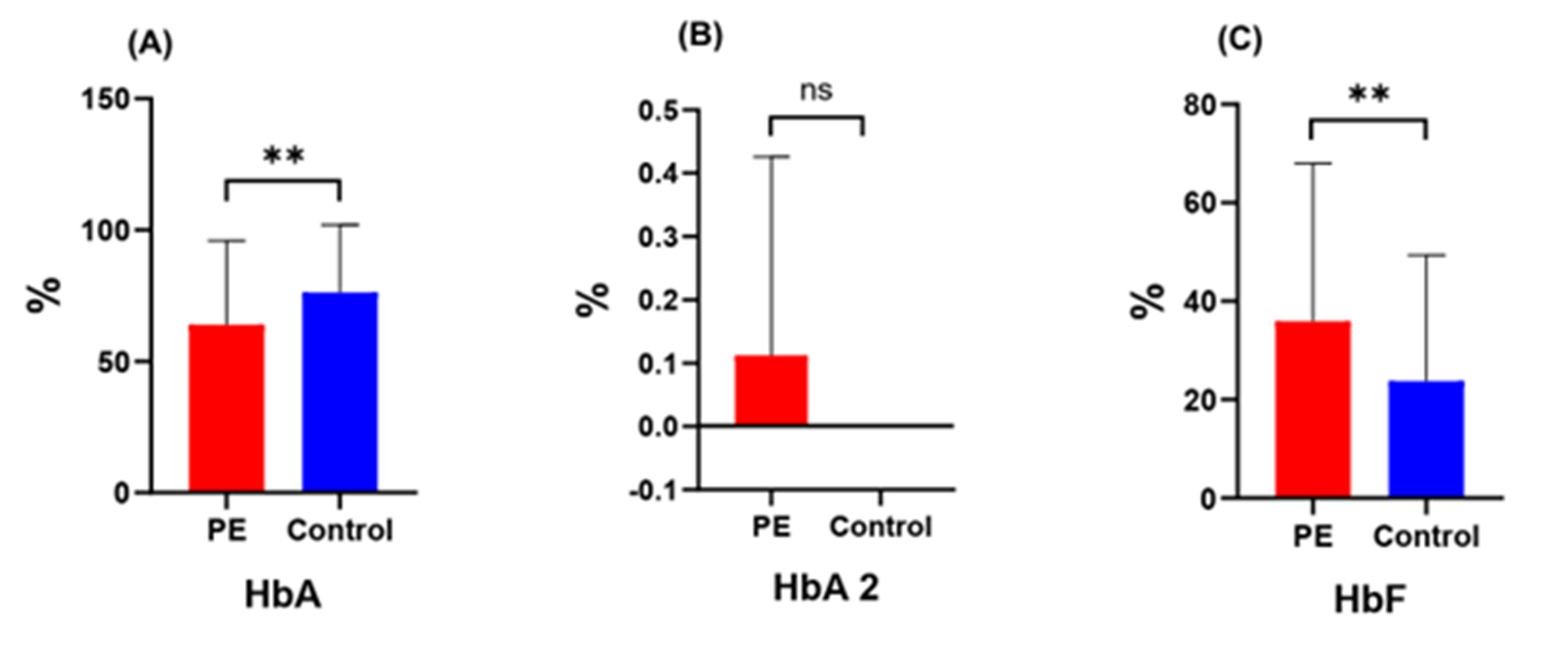

Supplement: S3 Fig — Hemoglobin electrophoresis analysis comparing the preeclampsia and Control groups. The concentration of HbA and HbF is significantly different between the two groups, while HbA₂ shows no significant difference. (TIF) [file pone.0324460.s005.tif]
